# Metabolic factors and post-traumatic arthritis may influence the increased rate of surgical site infection in patients with human immunodeficiency virus following total hip arthroplasty

**DOI:** 10.1186/s13018-020-01827-y

**Published:** 2020-08-12

**Authors:** Carol A. Lin, Phillip H. Behrens, Guy Paiement, W. David Hardy, James Mirocha, Robert L. Rettig, Heidi L. Kiziah, Andrew G. Rudikoff, Antonio Hernandez Conte

**Affiliations:** 1 Department of Orthopedic Surgery, Cedars-Sinai Medical Center, Los Angeles, CA USA; 2 Division of Infectious Diseases, Johns Hopkins University School of Medicine, Baltimore, MD USA; 3 Division of Biostatistics & Bioinformatics, Cedars-Sinai Medical Center, Los Angeles, CA USA; 4 Department of Surgery, Kaiser Permanente Los Angeles Medical Center, Los Angeles, CA USA; 5 Department of Anesthesiology, Kaiser Permanente Los Angeles Medical Center, 4867 Sunset Blvd, 3rd Floor, Suite 3017, Los Angeles, CA 90027 USA

**Keywords:** Human immunodeficiency virus (HIV), Total hip arthroplasty, Surgical site infection (SSI), Highly active antiretroviral therapy (HAART)

## Abstract

**Background:**

The impact of CD4+ T-cell count and highly active antiretroviral therapy (HAART) on the rate of surgical site infection (SSI) in patients with human immunodeficiency virus (HIV) undergoing total hip arthroplasty is still unclear. The goals of this study were to assess the rate of perioperative infection at a large tertiary care referral center and to identify risk factors in HIV+ patients undergoing total hip arthroplasty (THA).

**Methods:**

This study was a prospective, observational study at a single medical center from 2000–2017. Patients who were HIV+ and underwent THA were followed from the preoperative assessment period, through surgery and for a 2-year follow-up period.

**Results:**

Sixteen of 144 HIV+ patients (11%) undergoing THA developed perioperative surgical site infections. Fourteen patients (10%) required revision THA within a range of 12 to 97 days after the initial surgery. The patients’ mean age was 49.6 ± 4.5 years, and the most common diagnosis prompting THA was osteonecrosis (96%). Patients who developed SSI had a lower waist-hip ratio (0.86 vs. 0.93, *p* = 0.047), lower high density lipoprotein cholesterol (45.8 vs. 52.5, *p* = 0.015) and were more likely to have post-traumatic arthritis (12.5% vs. 0%, *p* = 0.008). Logistic regression analysis demonstrated that current alcohol use and higher waist-hip ratio were significant protectors against infection (*p* < 0.05). No other demographic, medical, immunologic parameters, or specific HAART regimens were associated with perioperative infection.

**Conclusions:**

Immunologic status as measured by CD4+ cell count, HIV viral load, and medical therapy do not appear to influence the development of SSI in HIV+ patients undergoing THA. Metabolic factors and post-traumatic arthritis may influence the increased rate of infection in HIV+ patients following THA.

## Background

Approximately 1.2 million people in the United States are living with human immunodeficiency virus (HIV) infection. Patients who are HIV-seropositive (+) encompass a wide clinical spectrum of disease with varying degrees of immunologic function [1]. Since the introduction of modern highly active antiretroviral therapy (HAART) in 1996, there has been a dramatic improvement in the survival of those who adhere to therapy [1–5]. While HAART has significantly improved the long-term prognosis of patients with HIV, improved longevity has also been accompanied by an increased risk of numerous comorbidities compared with the general population [6]. Atherosclerosis, dyslipidemia, diabetes mellitus, and lipodystrophy have all been described at higher incidences than in the general population in multiple HIV+ cohorts [7–10].

HIV+ patients are particularly at risk for the development of avascular necrosis of the femoral head, with many patients requiring total hip arthroplasty (THA) at a much younger age than those without HIV [11, 12]. Immunosuppression in the perioperative period and the occurrence of post-operative infections continue to be a major concern in this patient population [13, 14]. Early studies focusing upon this issue were conducted in the pre-HAART era [14], and additional studies were performed during the early HAART era, often including patients with hemophilia or intravenous drug use [15–17]. Furthermore, these studies were limited by small sample size [11] or lack of clinical follow-up [12, 18]. With the advent of newer, more effective HAART coupled with longer periods of medication adherence, the relationship between HIV and surgical site infection (SSI) is still unclear and can complicated by other viral infections or coexisting diseases [19, 20].

In this study, we sought to identify: (1) the post-operative rate of SSI for HIV+ individuals undergoing total hip arthroplasty (THA) at our institution, and (2) whether HIV+ related factors such as viral load and CD4+ T-cell count could predict postoperative infection.

## Methods

Patients who were enrolled in a prospective, observational study of HIV and cardiac risk at an urban academic medical center with an annual volume of >1000 total joint arthroplasty procedures per year were screened for inclusion. All HIV+ patients over 18 years old who presented for elective total hip arthroplasty between January 1, 2000 and March 15, 2017 were included in this study; patients under the age of 18 were excluded from this study. All patients were followed by an infectious disease specialist pre- and post-operatively with detailed immunologic and HAART records.

Detailed patient demographic information was collected at enrollment. Data points included smoking, drinking and recreational drug use history, past co-morbidity medical history, HIV-related history including acquisition risk factors and length of infection, HAART, and other medication history, immunologic laboratory parameters including CD4+ and CD8+ T-cell counts, CD4+/CD8+ ratio, and HIV-1 RNA level (viral load). Additional metabolic factors such as the presence of clinical lipodystrophy, laboratory testing for hyperlipidemia, low-density lipoprotein (LDL) and high-density lipoprotein (HDL) cholesterol levels, waist-hip ratio, and body-mass index (BMI) were also collected. Waist-hip measurement ratios are direct indicators of lipodystrophy in HIV+ patients on antiretrovirals.

All patients were followed for 2 years after the surgical procedure. Study patients were categorized into one of two groups: those who developed postoperative SSIs and those who did not develop SSIs (no SSI). The criteria for the diagnosis of a post-operative infection were based on the Centers for Disease Control Guidelines on organ/space SSI. The criteria were defined as follows: infection occurring within 30 days after the operative procedure or within 1 year if an implant was placed and appeared to be related to the original procedure; the infection involved any part of the anatomy, excluding the skin and area superficial to the fascia that was manipulated during the operation.

One of the following findings also had to be present: (1) purulent drainage from a drain placed through a stab wound into the surgical site, (2) micro-organisms isolated from an aseptically obtained culture of fluid or tissue, (3) clinical diagnosis of an abscess or other evidence of infection involving the surgical site found on direct examination, during re-operation, or (4) histopathologic or radiologic diagnosis of a SSI by a surgeon or attending physician [21].

Summary results are reported as mean ± standard deviation (SD) for continuous variables and frequency (percent) for categorical variables. Group (SSI versus no SSI) differences on continuous variables were assessed by the independent samples *t* test; if the variable distribution was not approximately normal in either group, the Wilcoxon rank-sum test was used to confirm. Group differences in categorical variables were assessed by the chi-square or Fisher exact test, as appropriate, depending on expected cell counts. Multivariable models were not used for the analysis of SSI with even two predictors as that may lead to over-fitting with the available data. With only 16 SSI events, models with at most 2 potential predictor variables were utilized. Logistic regression results are reported as odds ratios (OR) with 95% confidence intervals (CI). A two-sided 0.05 significance level was used throughout. Statistical analyses were performed using R version 3.3.2 (R Foundation for Statistical Computing, Vienna, Austria) and SAS version 9.4 (SAS Institute, Cary, North Carolina, USA).

## Results

Of the 144 patients who were enrolled, 16 (11%) developed perioperative infections, and 14 patients (10%) required surgical intervention between 12 and 97 days after the initial surgery (mean 42 ± 27). Patient mean age was 49.6 ± 4.5 years, and the most common pre-operative diagnosis for THA was osteonecrosis (95.8%). There were no patients with a history of coagulopathy. All patients had well-suppressed viral loads (undetectable or low-level detectable) and CD4+ T cell counts >200 cells/mm^3^. Similarly, all patients had been on HAART for more than 4 years with at least 90% compliance.

Tables 1 and 2 depict demographic, medical, and immunologic differences between those who developed an SSI and those who did not. Patients who developed infections had lower HDL cholesterol levels, lower waist circumference and were more likely to have post-traumatic arthritis. Both patients with post-traumatic arthritis had closed trauma (non-penetrating to joint) from the initial trauma, and they both developed a deep SSI requiring revision surgery within 45 days from the initial THA. Both trauma patients also had THA within 7 days of the initial traumatic event; the level of inflammation was not noted. Logistic regression modeling demonstrated that current alcohol use (OR 0.27; 95% CI 0.09–0.80, *p* = 0.014) and a higher waist-hip ratio (OR 0.54 for every increase in the ratio of 0.1; 95% CI 0.89–0.99, *p* = 0.049) were protective against SSI. No other demographic, medical social, laboratory, or immunologic factors were significant predictors of SSI.

**Table 1:** Demographic and medical history in patients with and without surgical site infection (SSI)

Variable ***n***	Category	No SSI 128	(+) SSI 16	***p*** value
**Age**		49.6 ± 4.5	50.2 ± 4.9	0.657
	Caucasian	117 (91%)	14 (88%)	
**Ethnicity**	Black	2 (2%)	2 (12%)	0.125
	Hispanic	8 (6%)	0 (0)	
	Asian	1 (0.8%)	0 (0)	
	Osteonecrosis	125 (98%)	13 (81%)	
**Indication for arthroplasty**	Osteoarthritis	3 (2%)	1 (6%)	**0.008**
	Trauma	0(0)	2 (12%)	**0.005**
**Drug Use**		66 (46%)	8 (50%)	1.000
**Smoking**		19 (13%)	1 (6%)	0.700
**Alcohol**		94 (65%)	9 (56%)	0.361
**Body mass index**		25.0 ± 3.7	24.9 ± 3.8	0.908
**Waist/hip ratio**		0.93 ± 0.09	0.86 ± 0.12	**0.047**
**Lipodystrophy**		80 (56%)	10 (63%)	0.792
**Hyperlipidemia**		43 (30%)	7 (44%)	0.266
**Total cholesterol (mg/dL)**		174.7 ± 31.5	166.2 ± 32.7	0.339
**HDL (mg/dL)**		52.5 ± 13.4	44.8 ± 10.7	**0.015**
**LDL (mg/dL)**		93.5 ± 25.8	84.4 ± 31.4	0.283
**Triglycerides (mg/dL)**		146.8 ± 81.4	185.6 ± 86.0	0.104
**Renal disease**		2	0	>0.99
**Hepatitis C**		2	0	>0.99
**Diabetes**		0	0	N/A

Continuous variables are presented as mean ± standard deviation.

Categorical variables are presented as number (percentage).

A *p* < 0.05 was considered significant

**Table 2:** Immunologic characteristics of HIV+ patients with and without Surgical Site Infection (SSI)

Variable ***N***	No SSI 128	SSI 16	***p*** value
**Years since HIV diagnosis**	15.9 ± 5.9	18.3 ± 5.1	0.086
**Mode of transmission (MSM)**	107 (84%)	12 (75%)	0.481
**Previous diagnosis with AIDS**	38 (40%)	8 (50.0%)	0.152
**Previous history of opportunistic infection**	29 (23%)	5 (31 %)	0.532
**Undetectable viral load**	118 (92.2%)	14 (87.5%)	0.624
**Compliance w HIV meds**	98.7 ± 2.2	99.4 ± 0.5	0.561
**Years on any HIV meds**	14.1 ± 5.1	16.5 ± 5.5	0.088
**CD4+ cell count**	615.9 ± 293.5	567.8 ± 178.0	0.746
**CD8+ cell count**	881.1 ± 315.8	866.9 ± 264.5	0.859
**CD4+/CD8+ratio**	0.77 ± 0.41	0.72 ± 0.33	0.729

Continuous variables are presented as mean ± standard deviation.

Categorical variables are presented as number (percentage).

A *p* < 0.05 was considered significant

## Discussion

This study represents the largest cohort of HIV+ THA patients with longitudinal follow-up and detailed immunologic data in the literature to date. We sought to determine the relationship between HIV infection, concomitant metabolic or comorbid medical conditions, virologic control of HIV (i.e., viral load) and immune status (i.e., CD4+ cell count, CD4+/CD8+ ratio)), and SSI following total hip arthroplasty. We found an infection rate of 11%, with nearly all of the cases presenting in the first 3 months following surgery. Immunologic restoration and concomitant antiretroviral therapy did not appear to be associated with the development of SSI and/or re-operation. However, on univariate analysis, patients who developed an SSI had significantly lower HDL cholesterol levels (44.8 ± 10.7 mg/DL), lower waist/hip ratio (0.86 ± 0.12), and a previous diagnosis of post-traumatic arthritis. Additionally, current alcohol use (>2 drinks per week) and a higher waist/hip ratio (0.93 ± 0.09) were found to be protective against infection by stepwise logistic regression analysis.

Prior retrospective series in HIV+ patients undergoing total joint arthroplasty (TJA) reported infection rates of 10–15%; however, many of these reports included patients with additional risk factors for infection such as intravenous drug use and hemophilia and the studies were conducted prior to the era of widely available HAART [14, 17, 19, 22, 23]. More recent studies have suggested that infection rates may be much lower [20]. Enayatollahi et al. performed a systematic review of 25 studies performed in the era of HAART therapy and analyzed a total of 722 TJA in HIV+ patients [20]. Issa et al. performed a retrospective cohort comparison of patients undergoing THA for osteonecrosis with a 10-year follow-up. Of the 44 THA performed, there were 2 late infections (5%), which was not statistically significant when compared with 0 infections in the HIV-negative group [24]. Similarly, Tornero et al. found only 1 infection (6%) in their series of 18 HIV+ patients undergoing THA [25]. They found that HIV+ patients without hemophilia had an overall infection rate of 2.3% compared with 10% in those with hemophilia. Furthermore, the presence of HAART was associated with a significantly lower risk of infection [20]. Given the low prevalence of HIV infection in the United States (~0.9% in general population) and difficulty in developing a sizeable cohort in any single institution, Lin et al. reported on the perioperative outcomes in THA using the National Inpatient Sample which represents a 20% sample of all inpatient admissions across the country [26]. The authors found that there was no difference in overall complication rates between HIV+ and HIV-negative patients. While there was a higher rate of wound infection and re-operation in HIV+ patients undergoing THA, regression analysis found that coagulopathy, malnutrition, and prior infection were more strongly associated with these infections than HIV itself [12]. Given these results, it seems possible that HIV alone might not be a risk factor for infection following TJA as much as associated comorbidities.

While no modern, comparative, longitudinal studies have conclusively shown a significant increase in infection following TJA, it may be due to limited sample sizes. Capogna et al. reported on 69 HIV+ TJA patients compared with 138 matched controls and found a 4% rate of infection, signifying a 6-fold increase infection risk [27]. Lin et al. compared 22 HIV+ TJA to 372 uninfected controls and found a 9% rate of infection in HIV+ patients compared with 2% for controls [11]. A post hoc analysis of their results found that a minimum of 135 HIV+ positive patients would be required to show a fivefold increase in infection [11]. An additional criticism of these series could also be that they analyzed both total knee and total hip arthroplasty together, which could have affected their results. While we did not follow a comparison cohort in our study, our sample size exceeds that of the necessary minimum calculated by Lin et al. Furthermore, we restricted our group to only primary THA patients which limits confounding variables and similarly found an 11% rate of infection, much higher than the reported SSI rate of 2% or less in primary THA [28].

There is conflicting evidence on the relationship between immunologic parameters and SSI in HIV+ patients. Guild et al. found that orthopedic trauma patients with HIV infection undergoing implant surgery were at increased risk of postoperative infection, especially those with CD4+ T-cell counts less than 300 cells/mm [29]. Other reports in the general surgery literature have found a CD4+ T-cell count <200 cells/mm^3^ to be associated with increased infectious complications [30, 31]. In contrast, multiple other studies have not found an association between CD4+ T-cell count and infection following surgery, with a wide range of preoperative CD4+ T-cell count values in patients with and without subsequent postoperative infection [11, 15, 32].

Viral load quantification has also been evaluated as a predictor of post-operative infection in HIV+ patients. Horberg et al. evaluated 372 matched pairs in the Kaiser Permanente database and found that an HIV-1 RNA level of 30,000 copies/mL or more was associated with an increased postoperative complication rate, although CD4+ T-cell count was not [15]. In contrast, King et al. found that that CD4+ T-cell counts were inversely associated with mortality following surgery; however, HIV viral load did not predict any complications [33]. In our study, all patients had been closely followed by an infectious disease specialist at a tertiary medical center and had been on HAART for at least 4 years prior to presenting for THA. The majority of our patients had undetectable viral loads, and, like prior studies, there was a wide range of CD4+ T-cell counts in both groups. Our study findings suggest that even in the optimized HIV+ patient, there appears to be a higher risk of SSI following primary THA compared with the general population.

As such, the development of SSI in HIV+ patients may be a more complex event related to factors other than HIV immunologic laboratory values. Prior studies have suggested that immunocompetence may be a dynamic factor in those patients who appeared well-managed prior to their implant surgery were found to have decreased CD4+ cell counts <500 cells/mm^3^ at the time an SSI was diagnosed. Alternatively, the change in immune status may have reflected a more global decline in overall health [11, 32]. Given that all of our study patients received care provided by infectious disease specialists at our institution, we were able to collect detailed metabolic data in addition to HAART and immunologic information. While we did not find a relationship between SSI and BMI or lipodystrophy, we did see an association between lower HDL cholesterol with an increased risk of SSI, which may be interpreted as a generalized metabolic indicator of health.

Long-term use of HAART has been associated with multiple metabolic and immunologic abnormalities such as dyslipidemia (total cholesterol >220 mg/dL), insulin resistance (glycosylated hemoglobin A1C >7%), endothelial dysfunction, and persistent, low-level inflammation (i.e., elevated C-reactive protein) [34–36]. The majority of patients in our study had some form of clinical lipodystrophy (56% no SSI; 63% with SSI) which can be preceded by insulin resistance as well as hyperlipidemia and may be evidence of long-term underlying metabolic changes. There is a strong association between hyperglycemia, diabetes, and SSI following orthopedic implant surgery [37–41], and it is possible that some underlying insulin resistance combined with the physiologic stress of surgery could have contributed to the development of SSI, despite the low rate of clinical diabetes mellitus. That patients who developed an SSI had lower HDL cholesterol levels, associated with increased cardiovascular risk and lower protections against inflammation [42–44], may also suggest that they may have more global metabolic changes that might impact immune competence compared to those patients that did not develop infections.

In contrast, the presence of a waist-hip ratio ≤0.85 is generally associated with improved cardiovascular health [45], and in our study, those who developed an SSI had an average waist-hip ratio of 0.86, which was significantly lower than those without an SSI on both univariate and logistic regression modeling. However, it is unclear if this lower waist-hip ratio in our study subjects with SSI was the result of anatomic rather than metabolic differences. It is possible that HIV+ patients with lower waist-hip ratios may have less adipose tissue over the hips at the surgical site. A possible hypothesis is that the surgical incision in these patients will be through a layer of poorly vascularized subcutaneous adipose tissue making wound complications and SSI more likely.

Current alcohol use was also found to be protective against surgical site infection with an estimated 50% reduction in risk. While heavy alcohol use has been associated with adverse cardiovascular events and postoperative infection [46–48], moderate alcohol use may have a beneficial effect by reducing inflammatory markers and improving vascular endothelial function [49–51]. Given the intensive medical and social optimization that is usually associated with preparation for elective THA, it is unlikely that those who reported current alcohol use were heavy users of alcohol but were more likely to be moderate users. As such, the association with decreased infection may be the result of beneficial modulation of inflammatory pathways from moderate drinking in contrast to and in a similar vein to the finding that HDL cholesterol was a marker of negative metabolic and inflammatory effects.

It is notable that all SSIs were diagnosed within the first 3 months (97 days) after surgery suggests that all recorded infections originated at the time of surgery. This again may be related to altered metabolic and inflammatory pathways. Surgery creates a pro-inflammatory, systemic response which could have unmasked underlying abnormalities in tissue healing, thus increasing the risk for SSI. For example, compromised soft tissues such as those scarred from prior surgery are associated with an increased risk of SSI following THA [52–54]. Furthermore, vascular endothelial function may potentially be altered by long-term HAART. This may compound the changes in local tissue perfusion caused by surgery impeding the local defenses against bacterial inoculation. The two patients with post-traumatic arthritis, notably, developed SSI requiring revision surgery. However, the SSI rate in our group would still be higher than the general population even if those patients were excluded from the cohort.

There are several limitations in this study which may limit broad generalizability. Although our patients were identified prospectively, our institution did not maintain an integrated electronic healthcare record for all of the years of the study population; therefore, this limited our ability to obtain detailed reports on implants, surgical time, intraoperative factors, and infectious organisms. Additionally, we were unable to collect functional outcomes data or extend the length of follow up beyond 2 years. Also, there was no HIV-negative comparison cohort to evaluate the possible effects of other comorbidities. Nonetheless, our study suggests that even in the well-managed, well-resourced, and otherwise medically optimized HIV+ patient, there is a significantly higher risk of surgical site infection than in HIV-negative patients.

Despite these limitations, our study reports on the largest, prospectively followed, homogenous cohort of HIV+ patients undergoing elective primary THA with detailed immunologic data. Our data suggest that even in well-managed, well-resourced, medically optimized patients living with HIV, we may anticipate a higher rate of risk for SSI than that reported in the general THA population (2% or less) [28, 55]. Based on these results, it does not appear that the risk of SSI in these patients is associated with any particular immunologic laboratory value or associated medical comorbidity but may be explained by subtler and more complex metabolic and inflammatory changes that occur with a long-term history of HIV infection and its associated treatment.

## Conclusions

HIV+ patients and the physicians providing care for them should be aware of the possible interactions between HAART, metabolic changes, vascular endothelial changes, and increased surgical site infection following THA. Predicting the incidence of infection in HIV+ patients undergoing hip arthroplasty remains a complex issue and close surveillance should be maintained in this patient population.

## Data Availability

The datasets generated and/or analyzed during the current study are not publicly available due to strict HIV confidentiality requirements by our Institutional Review Board but are available from the corresponding author on reasonable request.

## Abbreviations

HAART: Highly active antiretroviral therapy
HIV: Human immunodeficiency virus
SSI: Surgical site infection
THA: Total hip arthroplasty
